# Feasibility and application of machine learning enabled fast screening of poly-beta-amino-esters for cartilage therapies

**DOI:** 10.1038/s41598-022-18332-3

**Published:** 2022-08-20

**Authors:** Stefano Perni, Polina Prokopovich

**Affiliations:** School of Pharmacy and Pharmaceutical Sciences, Cardiff University, Redwood Building, King Edward VII Avenue, Cardiff, CF10 3NB UK

**Keywords:** Drug discovery, Chemistry, Mathematics and computing

## Abstract

Despite the large prevalence of diseases affecting cartilage (e.g. knee osteoarthritis affecting 16% of population globally), no curative treatments are available because of the limited capacity of drugs to localise in such tissue caused by low vascularisation and electrostatic repulsion. While an effective delivery system is sought, the only option is using high drug doses that can lead to systemic side effects. We introduced poly-beta-amino-esters (PBAEs) to effectively deliver drugs into cartilage tissues. PBAEs are copolymer of amines and di-acrylates further end-capped with other amine; therefore encompassing a very large research space for the identification of optimal candidates. In order to accelerate the screening of all possible PBAEs, the results of a small pool of polymers (n = 90) were used to train a variety of machine learning (ML) methods using only polymers properties available in public libraries or estimated from the chemical structure. Bagged multivariate adaptive regression splines (MARS) returned the best predictive performance and was used on the remaining (n = 3915) possible PBAEs resulting in the recognition of pivotal features; a further round of screening was carried out on PBAEs (n = 150) with small variations of structure of the main candidates from the first round. The refinements of such characteristics enabled the identification of a leading candidate predicted to improve drug uptake > 20 folds over conventional clinical treatment; this uptake improvement was also experimentally confirmed. This work highlights the potential of ML to accelerate biomaterials development by efficiently extracting information from a limited experimental dataset thus allowing patients to benefit earlier from a new technology and at a lower price. Such roadmap could also be applied for other drug/materials development where optimisation would normally be approached through combinatorial chemistry.

## Introduction

Biomaterials and drug design are regarded as a very resource (physical, economical and time) intensive operations^1^; the process can be constructed into sequential stages (discovery, preclinical, clinical and pharmacovigilance) named Phase0 to Phase4. During Phase0, traditional bench experiments are carried out to identify optimal candidates that are screened through further developmental stages; while further clinical trials progressively assess toxicity, efficacy and long term safety (Phase1to Phase4)^2^. The overall development process can take from a minimum of 5 up to 15 years with an estimated total development cost per approved drug of $2168 million in 2018^3^. However, the actual costs are generally a commercial confidential information and, therefore, such estimates may not fully capture the complete investments required^4^. The try-and-error approach to molecule development, particularly during the initial design and make phases of the design-make-test-analyse (DMTA) discovery cycle, is often directed by human intuition, which is inherently biased and limited in knowledge, thus slowing drug development^5^. In such contest, the ability of data-driven in-silico prediction tools to model outcomes without the need to physically prepare candidates and run experiments would enable a fast throughput screening of candidate molecules and thus reducing both the time and monetary investments required to identify lead candidates^6–9^. This can be achieved by establishing correlations between certain properties of the molecules (inputs, also known as descriptors) and outcomes of interest using experimentally generated data on a subset of relevant compounds; the established model would then be used to predict outcomes on the wider molecule search space^10^.

**Figure 1:**
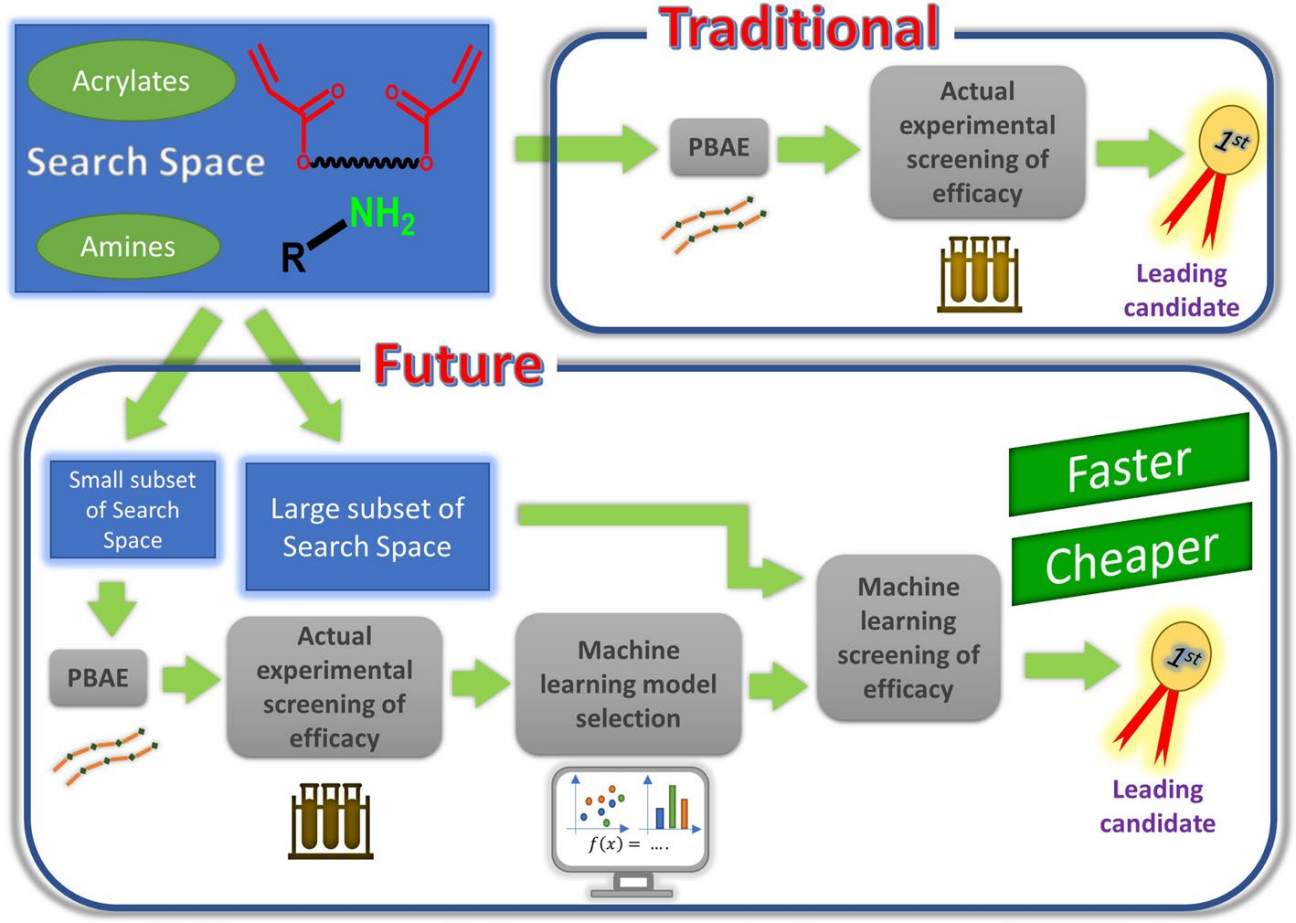
Schematic representation of machine learning driven drug development process.

Machine learning (ML) based regression techniques are becoming wide spread in many areas of data analysis in the chemical^11,12^ and pharmaceutical sector^13–16^; they have recently been employed in drug development^17–19^, diagnostic^20^, treatment algorithm optimisation^21^, drug repurposing^2,22^ and material discovery^23,24^; however such applications are still quite limited despite being very promising^25,26^. Another application of ML technologies in drug discovery is during compound screening or hit/lead generation and optimization enabling a virtual screening platform that offers a quicker and cheaper alternative to classic testing of large compounds libraries^27,28^; virtual screening can be generally classified in ligand-based or structure-based^28^. Compound optimisation using ML enabled virtual screening has been successfully applied to drug development for Alzheimer’s disease^29^, Class B G protein-coupled receptors (GPCRs)^30^ and antiviral^31^. Figure 1 depicts how ML could be deployed to accelerate the biomaterial development process through virtual screening. Despite the flexibility of ML techniques, material design and optimisation involving numerous parameters are situations more likely to benefit from the development of machine learning predictive models.

Osteoarthritis (OA) is a thinning or loss of the cartilage layer covering the surfaces of joints reducing articular mobility, causing pain and inflammation. Although OA is not a life threating disease, it has a great impact on the quality of life of patients and their ability to perform regular activities resulting in a great burden to society and health care providers. Worldwide, 303.1 million of people live with hip or knee osteoarthritis^32^; furthermore, OA prevalence is expected to grow as consequence of the ageing population and overnutrition (two critical risk factors for OA). An effective treatment is still missing, current therapies (anti-inflammatory and analgesics) are only managing symptoms. This lack of therapeutic options is compounded by the inability of delivering the active molecule where is needed because of the obstacles posed by the low vascularisation and high electrostatic repulsion of cartilage tissues; these factors limit the amount of drug effectively available to the targeted cells^33^. In order to achieve drug localisation, without a delivery system, high concentrations of drugs are used in the synovial fluid as mass transfer is governed by concentration differences (Fick’s law)^34–36^. Such approach has some problematic drawbacks; firstly, it is a wasteful use of the drug as only a minimal amount is actually therapeutic, with consequences on treatment acquisition costs. Secondly, drug washout lead to systemic exposure with possible side effects, as in case of steroids^37^.

Different drug delivery systems have been developed for the localisation of drugs in cartilage in the attempt to overcome such barriers; poly-beta-amino-ester (PBAEs)^38,39^ and avidin^34^ are two examples of these delivery systems. While no particular optimisation of the delivery system based on avidin performance is feasible as this a well-defined protein; there are, instead, essentially ∞^2^ possible PBAEs as these are copolymers of an amine and a di-acrylate^40^. Moreover, when PBAEs end-capping is also considered, the possible combinations rise to ∞^3^. In light of the performance of PBAE as cartilage drug delivery system being extremely dependent on the polymer backbone; ML algorithms predicting the efficacy of the drug delivery in cartilage from the polymer’s constituents’ properties would provide a high throughput screening for the optimisation of the PBAE driven cartilage drug localisation technology, reducing the cost and time to select the most promising candidate. We have previously demonstrated how the uptake of dexamethasone (DEX) (a drug routinely administered in clinics through intra-articular injections to reduce OA symptoms) in cartilage tissue, through a poly-beta-amino-ester drug delivery system, could be modelled using partial least square regression^38,39^. The inputs of this model are the physical properties of the polymers and co-polymeric units (di-acrylate and amine) along with some experimentally obtained parameters such as the diffusion coefficient of the polymer through cartilage, the drug loading in the delivery system and the molecular weights (Mw and Mn) of the polymer chain^39^. Through this previous work, we identified a polymer (current lead candidate obtained from screening the combination of 3 acrylates and 15 amines) that increased DEX uptake in cartilage about 8 times compared to the clinical formulation^39^. Despite the ability of predicting uptake, this model, in order to make predictions on new candidates, still requires inputs generated by experiments (such as Mw, Mn and diffusion coefficient) thus not fully able to completely substitute lab-based work. With the purpose of accelerating the optimisation of the PBAE structure for the cartilage delivery system through a systematic screening of a large library of both acrylates and amines, we hypothesised that machine learning algorithms, utilising only predictors available in public libraries or calculated from the compound structure, namely the physico-chemical properties of the PBAE components, could be employed to fully predict the performance of the delivery system without the need for any experimentally originated data. Drug uptake data experimentally obtained from a subset of a large polymer library were utilised to train and optimise 25 machine learning models (e.g. Random Forests, Kth nearest neighbour (kNN), support vector machine (SVM), neural network and multivariate adaptive regression splines (MARS)) and investigated their predictive performance to identify the most accurate algorithm. This model was then employed to screen the PBAEs research space (round1) representing acrylates and amines with a wide range of structural features and moieties; key features in the amine and acrylate structure were recognised in the PBAEs predicted to return the greatest drug uptake, further elucidating correlations between PBAE structural properties and drug uptake. A further round of ML predictions (round2) was conducted to refine and improve efficacy, screening a new set of PBAE exhibiting structures with small variations of the core features of the main candidates identified in the first round. The most promising candidate identified at the end of round2 had a predicted 3 folds efficacy improvement over the previous best performing candidate (round1). Finally, the actual efficacy and safety of the predicted best candidate were also experimentally determined.

## Results

### Machine learning model selection

**Figure 2:**
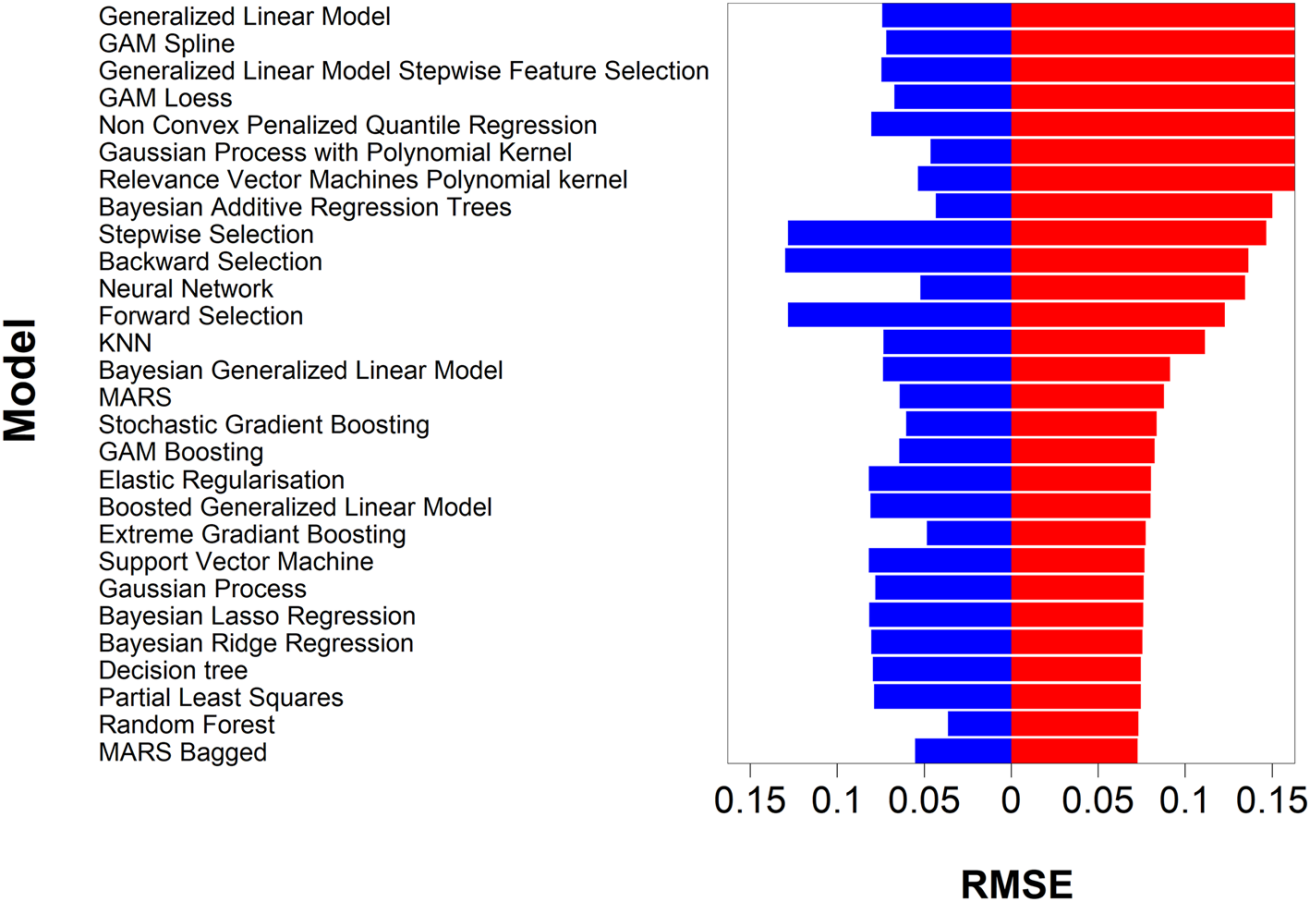
Comparison of the different performance of the tested algorithms on the train (blue) and test set (red).

Amine 1 to 20, acrylates A to F and end-capping e-1 and e-2 were used to generate the library of PBAE-DEX used for the experimental determination of DEX uptake in cartilage; in total 15*6*2 = 180 unique PBAE were synthesised, doubling the size of the experimentally tested PBAE. After random splitting, the train set included 70 PBAEs, while the remaining 20 PBAEs constituted the test set. As the ultimate purpose of modelling is being able to estimate outcomes (in our work the uptake of DEX in cartilage) on previously unseen predictors, a split of the initial dataset into train and test set was implemented to be able to identify the model with the greatest predicting ability that is not necessarily the one that return the most accurate fit of the data used to calculate the model parameters (i.e. regression coefficients)^41,42^. For the same reason, data split in training and test set was stratified based on PBAEs thus experimental data of DEX uptake for different exposure duration and related to PBAE with different end-capping all belonged to one set only. The 25–75% split also is in the typical range to provide sufficient data points for both model parameters estimation (training set) and testing^43–46^. Therefore, it was expected that all models performed better on the training set than on the test set (Fig. 2).

Bagged multivariate adaptive regression splines (bagged MARS) returned the lowest Root Mean Squared Error (RMSE) on the test set (0.072). Random Forest had the lowest RMSE on the training set (0.036) but the second lowest on the test set (0.073). Furthermore, regressions based on decision trees/random forests do not allow for extrapolation of the measured outcome beyond the training set and such would limit the possibility of identifying PBAE performing better the experimentally observed optimal candidate. Linear regression (forward, backward or stepwise) had the highest RMSE on the training datasets, 0.128, 0.128 and 0.124, respectively. The difference in model performance between train and test set depended on the algorithm used; for example elastic regulation had RMSE of 0.080 and 0.081 for train and test set respectively, while Bayesian addictive regression trees returned RMSE of 0.043 and 0.149 on train and test set, respectively. The small difference between the RMSE on train and test set observed for the elastic regulation model is a consequence of the penalties assigned to predictors in the algorithm that reduce the risk of overfitting^42,47^. Moreover, boosting and bagging improved model performance (Fig. 2), for example RMSE of bagged MARS was lower than MARS and random forests had lower RMSE than decision tree. This was expected as such approaches have been developed to improve on model performance^42,47^. Bagging is the process of resampling from the same data set to generate numerous new datasets then used to fit the model, this bootstrapping reduces overfitting and model variability^42,47^; on the other hand, boosting employs weak predictors to improve on the predictions of other predictors^48^.

**Figure 3:**
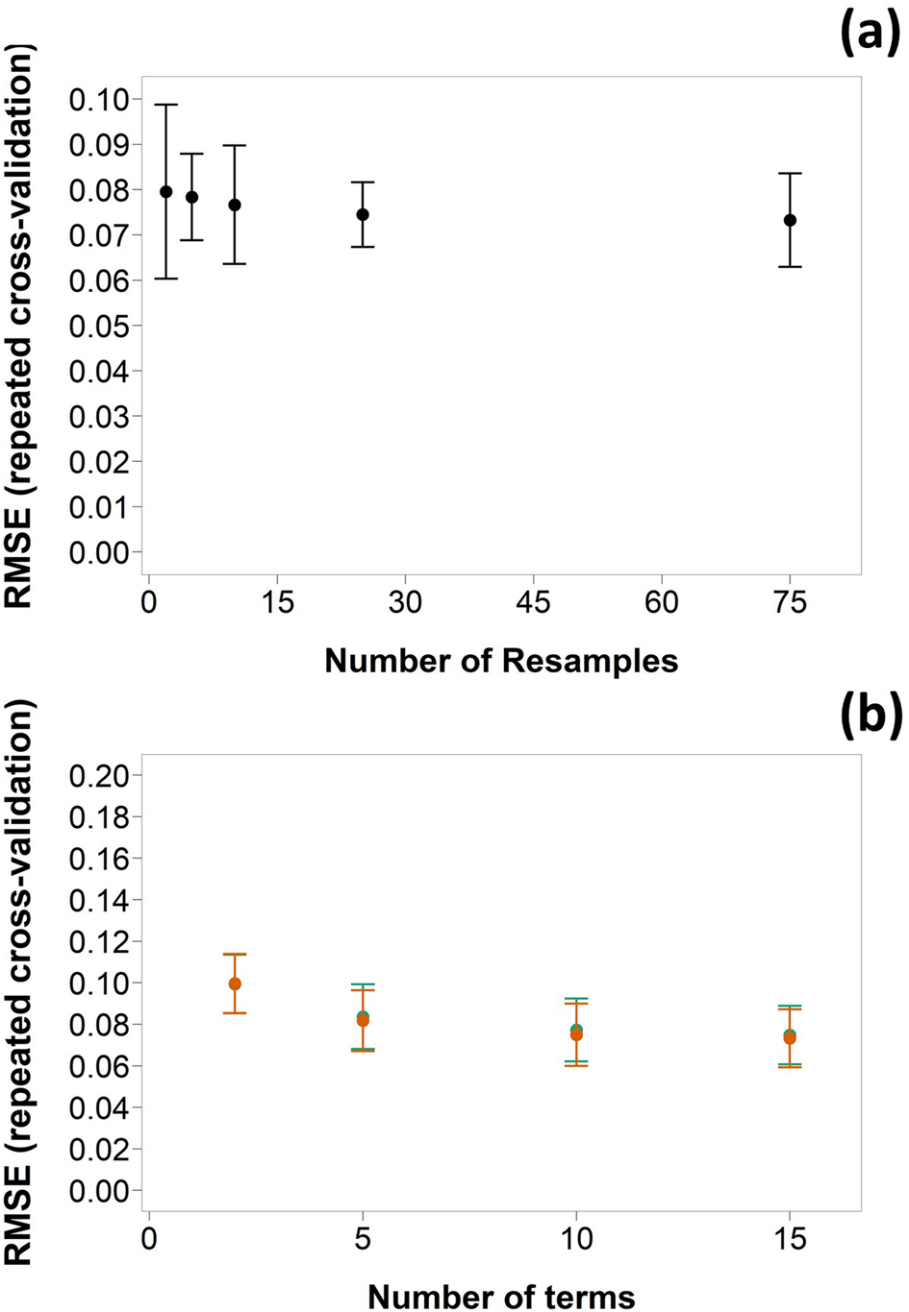
Relation for bag MARS models between RMSE (mean ± SD) for tenfold cross validation repeated 3 times and (**a**) number of resamples and (**b**) number of terms and degree of correlation (n = 1 , n = 2 ) (number of resamples = 75).

The optimisation of the bagged MARS model hyper-parameters showed that with increasing number of bagged samples, mean RMSE during cross-validation decreased; averaging 75 resamples gave the lowest RMSE (Fig. 3a) while the number of pruned parameters increased model performance monotonically, but RMSE marginally decreased with the combinations of more than 10 (Fig. 3b). Moreover, performance of bagged MARS improved when the degree of interaction between parameters increased from 1 to 2 (Fig. 3b). The optimal bagged MARS model was made of a combination of 75 MARS models with a median number of predictors of 9 and a median number of terms of 15.

**Figure 4:**
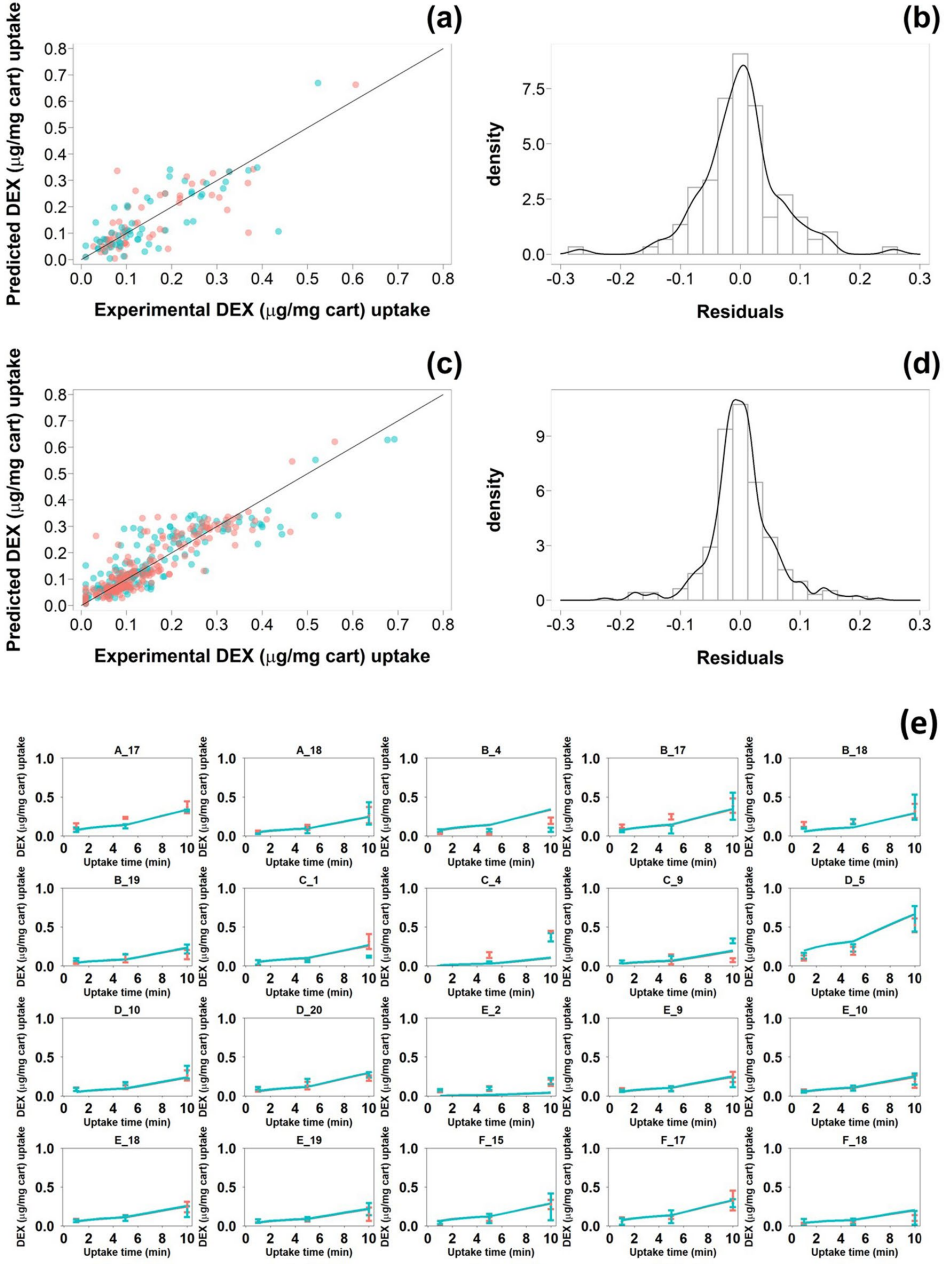
Comparision of predicted and experimental DEX uptake for PBAE-DEX (endcapped with e-1  and e-2 ) in the test (**a**) and train set (**c**); distributioin of residuals of DEX uptake predictions for PBAE-DEX in the test (**b**) and train set (**d**). Comparison of time dependent DEX uptake (mean ± SD) in cartilages predicted by optimised bag MARS model for PBAE in the test dataset (**e**).

DEX uptake predicted by the optimised bagged MARS model against the actual data for the test data set (Fig. 4a) revealed a general good agreement between prediction and actual data regardless of the PBAE end-capping agent while the residual distribution exhibited a gaussian distribution (Fig. 4b). Similar patterns were observed when the model was applied on the train set (Fig. 4c and d); however, the residuals were smaller resulting in a narrower distribution. Modelled uptake curves of DEX in cartilages with PBAE in the test set demonstrated a general good fit of the experimental data (Fig. 4e).

**Figure 5:**
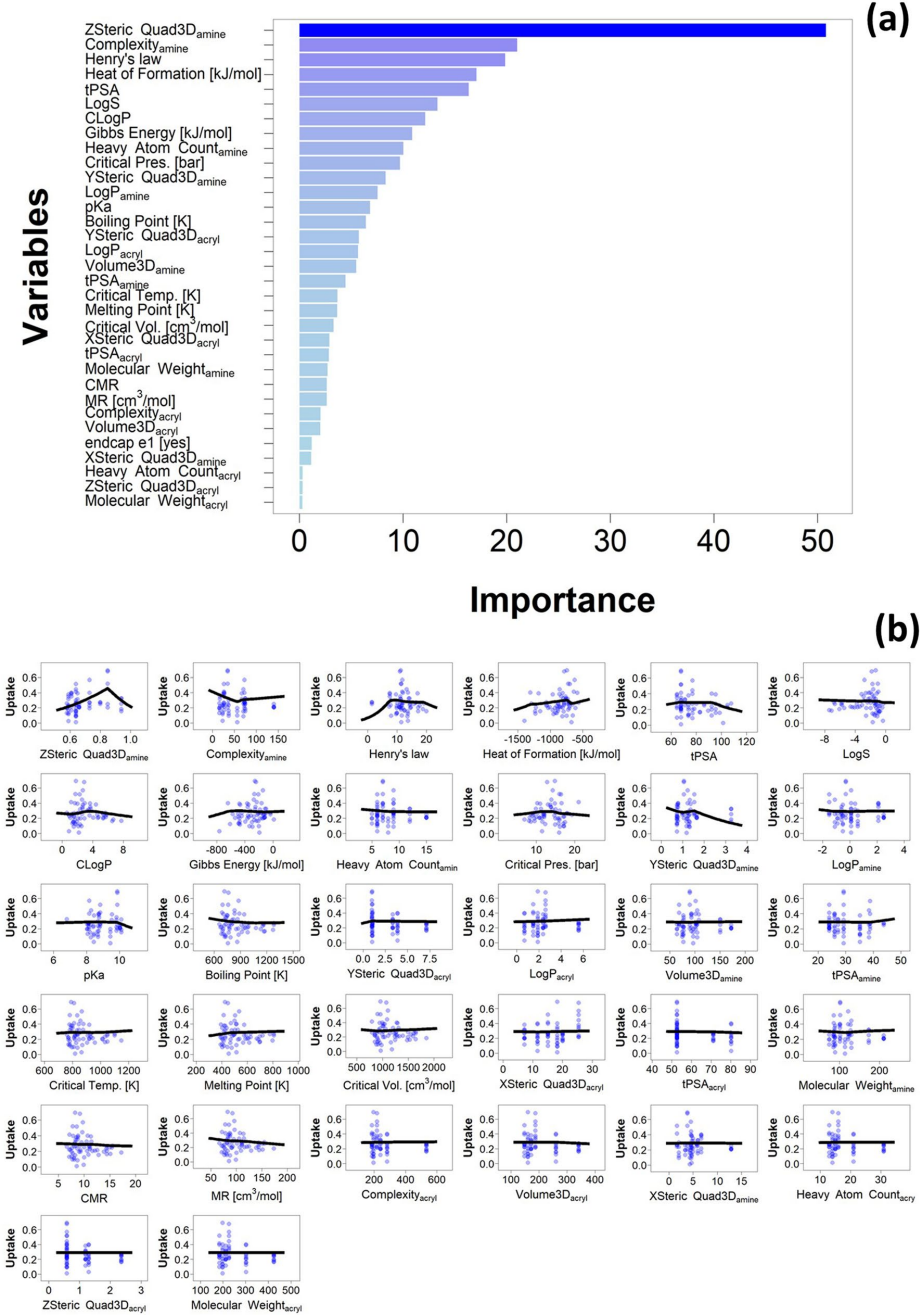
Variable importance in optimised bagged MARS model (**a**) and partial dependency plot of optimised bagged MARS model compared to experimentally obtained data for 10 min uptake of DEX into cartilage (**b**).

The variable with the greatest importance in the bagged MARS model was ZStericQuad3D of the amine component, followed by the complexity of the amine component and the Henry’s law coefficient of the PBAE repeated unit; the variable with the lowest importance was the molecular weight (MW) of the acrylate component (Fig. 5a). In order to gain insights on the relations between the chemical and topological properties of the PBAE and the efficacy in localising DEX in cartilage, the specific dependence of the DEX uptake on the individual predictors was analysed on through the partial dependency plot (PDP).

These plots represent the predicted outcomes against a single varying input variable while maintaining the remaining constant at their mean values. PDP revealed ZStericQuad3D returned a maximum DEX uptake at ~ 0.83; while complexity of the amine decreased DEX uptake for values up to 50, for greater amine complexity predicted DEX uptake increased monotonically but was lower than the maximum (complexity = 0) for the maximum amine complexity in the library tested (Fig. 5b). As the models were trained on transformed values the relations between variables and drug uptake does not appear linear on the back-transformed predictions.

### PBAE structure optimisation

**Figure 6:**
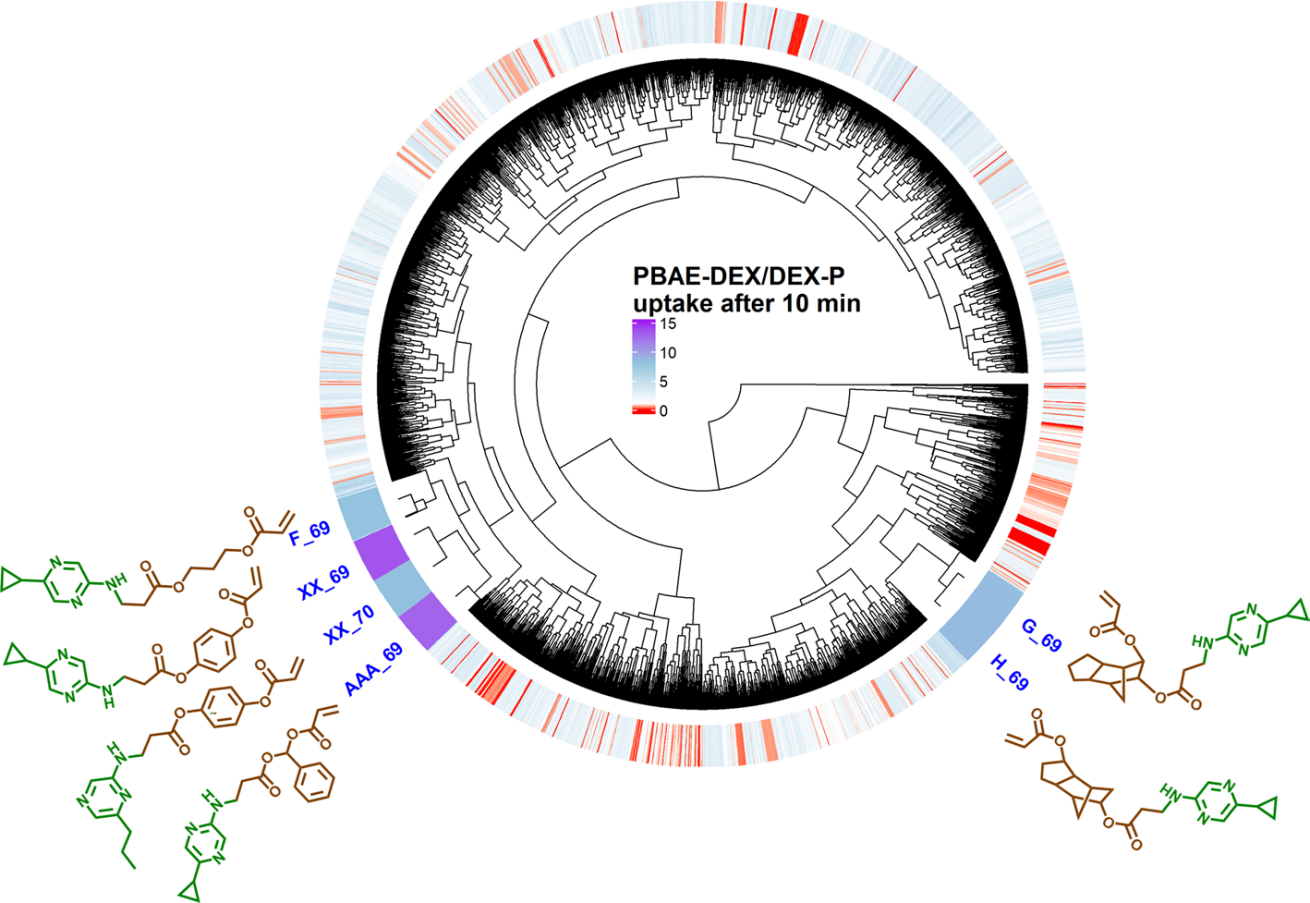
Heatmap of predicted ratio of DEX uptake for PBAE endcapped with e1 conjugated with DEX over commercial formulation of DEX after 10 min of exposure and structure of PBAE repeated unit with predicted drug uptake superior to experimental found candidate.

The optimised bagged MARS model was applied on the remaining PBAE search space constituted by 3915 un-synthesised polymers to predict DEX uptake in cartilage after 10 min of exposure to PBAE-DEX when end-capped with e-1 or e-2. The results of this round1 screening identified 3192 PBAEs, regardless of the end-capping (end-capping agent e-1 returning predominantly higher drug uptake than e-2 on the same PBAE backbone), with an expected DEX uptake greater than the commercial formulation. Furthermore, 11 polymers with a predicted uptake greater than the previous leading candidate, which returned a drug uptake about 8 times that of DEX commercial formulation, were identified through the model. These PBAEs clustered very closely according to the dendrogram determined using the chemico-physical properties of the polymers and were made mainly by acrylate AAA (Phenylmethanediol diacrylate) or XX (1,4-Phenylene diacrylate) and amine 69 (2-Amino-5-(cyclopropyl)pyrazine) or 70 (2-Amino-6-propylpyrazine). PBAE candidate XX-69 was predicted to exhibit the greatest uptake among the full PBAE library tested, about 13 folds greater than the commercial formulation (Figs. 6 and S5).

**Figure 7:**
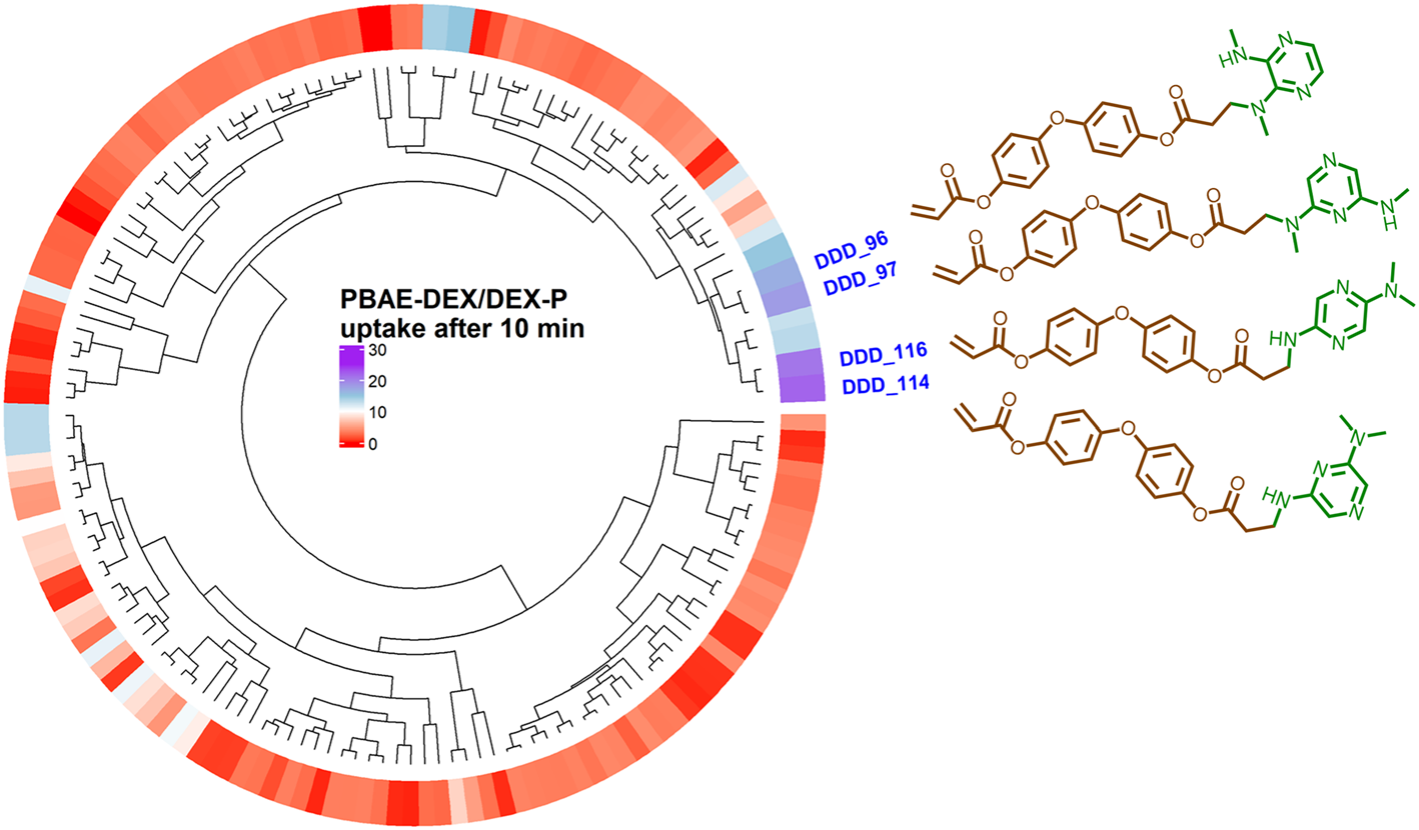
Heatmap of predicted ratio of DEX uptake for PBAE during round2 endcapped with e1 conjugated with DEX over commercial formulation of DEX after 10 min of exposure and structure of PBAE repeated unit with predicted drug uptake superior to best candidate in round1.

1,4-phenylene diacrylate and (acrylate XX) and phenyl-methanediol diacrylate (acrylate (AAA) are the only acrylates tested exhibiting a benzene group where the electron of the oxygen atoms forming the di-acrylate groups can resonate reducing the impact of the electrostatic repulsion between some areas of the PBAE backbone and glycosaminoglycans (GAG) constituents of cartilage. Similarly, the presence of pyrazine in the amine constituent can increase the availability of the electron pair in the nitrogen resulting in higher positive charge. These two features were assumed to be key properties for effective drug delivery in cartilage and a refinement of the PBAE structure was carried out screening further acrylates (n = 3) exhibiting at least a benzene group in proximity of the acrylate moiety along with amines (n = 50) with a pyrazine in their structure (Fig. S6) in Round2. 17 of the 150 PBAEs tested in round2 had an estimated DEX uptake greater than XX-69 (best performer in round1); the presence of a further tertiary amine bound to the pyrazine ring resulted in greater DEX uptake in cartilage; moreover, two benzine groups (Bisacrylic acid oxybis(4,1-phenylene) ester) improved on the drug delivery (Figs. 7 and S7). The most effective PBAE (DDD-114) identified in round2 had a predicted DEX uptake about 21 time greater than the commercial formulation.

### Ex-vivo performance of best candidate

**Figure 8:**
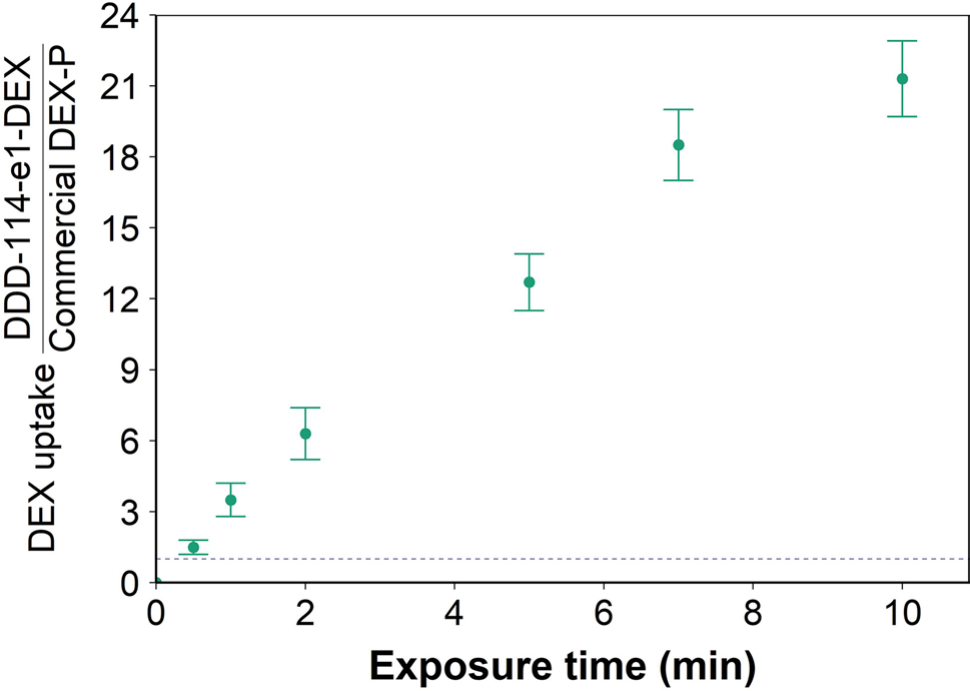
Uptake profile of DEX in cartilage using predicted best performing PBAE (DDD_114_e1-DEX).

DEX uptake in cartilage using DDD_114 increased with increasing exposure time; after 10 min the among of drug retrieved from the samples using the PBAE based drug delivery system was over 20-folds the commercial DEX-P formulation confirming the model predictions (Fig. 8).

**Figure 9:**
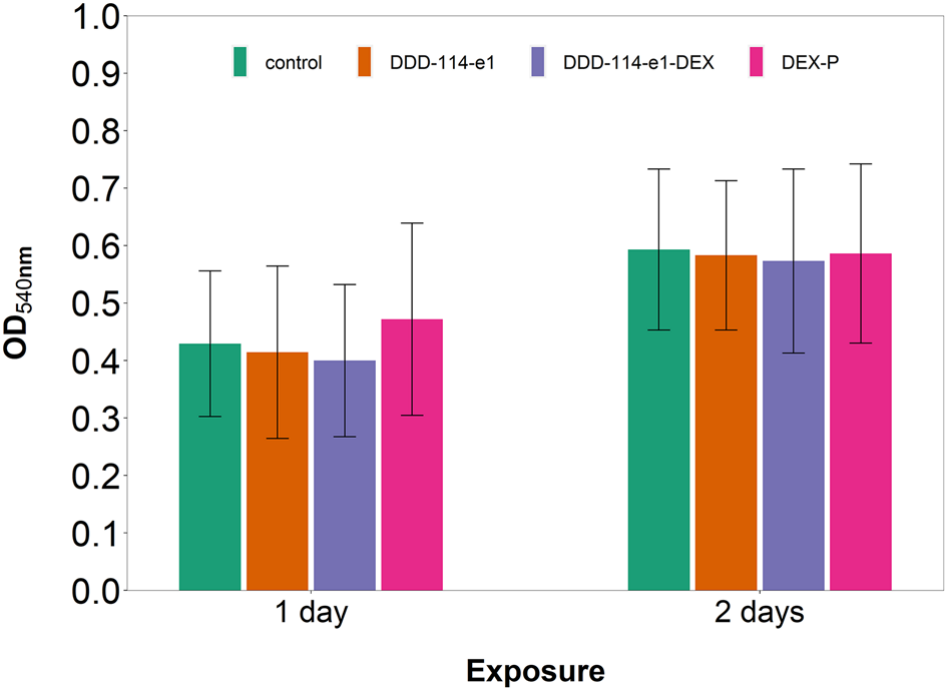
Mitochondrial activity of chondrocytes in cartilage explants cultured with basal media or medium containing predicted best performing PBAE (DDD_114_e1-DEX).

Mitochondrial activity of chondrocytes was not affected by the presence of the polymer (DDD_114_e1) (*p* > 0.05) (Fig. 9).

## Discussion

The key to accurate predictions through mathematical models is the size of the data set used for the estimation of the model parameters^49^. As our previous work hinted to the possibility of modelling cartilage drug uptake achieved by PBAEs conjugated to DEX^39^, the machine learning models in this work were trained using a dataset^39^ doubled in size with further polymers to reach a sufficient level of confidence in the model estimates. The work presented here considers only two end-capping agents treated as a categorical variable; the actual properties of the compounds were not considered as the number of molecules did not allow to capture such parameters.

Majority of research dedicated to implement ML in drug discovery/chemistry employs a very narrow range of potential models, even just one^49–51^, without a clear rationale for the selection of the algorithms included in the pool assessed^5,18,52–55^. Here instead, we purposely screened a large number of potential algorithms based on different approaches (e.g. decision tree, linear regression, SVM and neural network) in order to maximise the strength and transferability of the results while, simultaneously, increase the likelihood of identify a satisfactory predictive model.

MARS are an extension of linear models that can account for nonlinearities between input and output values through the use of hinge functions and interactions between variables combining flexibility and interpretability of results^42,47^. The overall regression model “goodness of fit” depends on hyperparameters such as the number of pruned parameters and the degree of interaction between predictors. Bagged MARS is an ensemble of MARS constructed on a randomly generated bootstrapped set of data. Although it was expected that aggregating further resamples would improve model predictive performance, no more than 75 resamples were implemented in this work as the reduction in RMSE from 50 to 75 resamples was already minimal and a further increase of the resamples would also impact computational time.

The efficacy end-point experimentally assessed in this study was the amount of drug retrieved from a cartilage sample after contact with the PBAE based delivery system; this could not itself differentiate between actual drug penetration or accumulation on the external cartilage surface. However, previous evidence demonstrated that PBAE drug delivery systems diffuse inside the cartilage tissue underlying the validity of the approach^38,56^. The electrostatic interactions between positively charged PBAE and cartilage tissue components (predominantly the highly negatively charged GAGs) are the key mechanism of action of the delivery system under the presented investigation. The ranking of the PBAE properties variables showing quadrupole on the Z axis of the amine component as the key parameter demonstrated by the analysis of variable importance is in agreement with the mechanisms of action and it was also found to be one the key parameters when PLS regression was carried out using not only chemico-physical properties but also experimentally determined characteristics (diffusion coefficient, zeta potential and molecular weight of the polymer)^39^. These PBAEs properties were not explicitly considered in the work as it was assumed that they depend on the properties of the amine and acrylate constituents and that the ML models would capture the correlation between drug uptake and polymer properties such as MW implicitly.

The optimal components identified here are structurally very different from those found as optimal copolymers for PBAE application in DNA vector^40,57^ and a direct consequence of the different mechanisms involved in the technology (DNA binding and cell membrane penetration vs. electrostatic attraction toward negatively charged GAG chains in cartilage).

The application of ML to PBAE structure optimisation for drug delivery in cartilage presented in this work can also potentially act as blueprint for the optimisation of other applications of PBAE such as drug releasing degradable coatings^58^, non-viral DNA vectors for gene therapy^40^ and mRNA vaccines^57^ fast-tracking products to patients where, to date, only a lab based combinatorial chemistry approach to optimisation has been undertaken^59^. The expected reduction in the time required to screen numerous polymers will also be coupled with monetary saving in the drug development costs with clear benefits not only to patients but also to health care providers.

We demonstrated an ML guided drug design optimisation approach that accurately predicts the relation between structure/property and outcome requiring only 2% of the compositional space (90 out of at least 3915 copolymers) to be experimentally explored. Our work led to the discovery of several PBAEs expected to result in a higher drug uptake than those of previously reported candidates. The actual efficacy was also determined and found to be very close to that predicted by the model (Fig. 8). Additionally, no negative impact on chondrocytes viability (Fig. 9) was detected for such PBAE as in line with the well-known safety of such polymers^40,59^. Moreover, the trends uncovered between properties and efficacy of the polymers, along with the non-intuitive optimal design elements of PBAE for cartilage delivery identified in this study, such as the presence pyrazine in the amine constituent (likely related to the increased hydrogenation of the nitrogen atom), are also critical in the search for next-generation polymer driven cartilage delivery systems.

## Methods

PBAE are denoted throughout the text with a code containing letters referring to the diacrylate (Fig. S1) and numbers (Fig. S2) referring to the amine; for example, A5 is the polymer made from 1,4-Butanediol diacrylate and 3-(dimethylamino)propylamine. The polymer backbone code is followed by e1 for PBAE end-capped with ethylene-diamine and e2 for PBAE end-capped with diethylene-triamine.

### Data analysis

All models were fitted through R^60^ and all other necessary packages necessary to perform regression with the “caret” package^61^.

Manhattan distance between PBAEs and complete distance between clusters were used for generating dendrograms.

#### Datasets and descriptors

Two PBAE uptake datasets were used to develop predictions, the publicly available set^39^ was expanded with a purposely obtained new set collected after the inclusion of further acrylate monomers in the library.

Drug uptake predictions were performed utilizing physical and chemical parameters of amine and acrylate components of each PBAE obtained from PubChem library (Mw, logP, tPSA, Complexity, Heavy Atom Count, Volume 3D, X_Steric Quadrupole 3D, Y_Steric Quadrupole 3D, Z_Steric Quadrupole 3D); along with parameters related to the repeated polymeric unit (amine + acrylate) calculated through ChemDraw. The later included boiling point, melting point, critical volume and pressure, Gibb's free energy, logP (partition-coefficient between two immiscible phases at equilibrium which is proportional to hydrophobicity), solubility (logS), pKa, molar refractivity (CMR), heat of formation and the topological polar surface area (tPSA), which represent the total area of all polar atoms (mainly oxygen and nitrogen) including their affixed hydrogen atoms.

Kth nearest neighbour imputation was employed to handle missing data^47^.

#### Machine learning algorithms training and predictions

Outcome data were transformed (1/y^4^) to achieve a distribution of the drug uptake closer to a gaussian profile; moreover, input values for possible predictive variables were centred and scaled using mean and standard deviation.

A random split of the PBAEs into training (75%) and test (25%) datasets was applied. Weights to each point were assigned proportionally based on the distance from the median. Classic Machine Learning methods, such as Bernoulli Naive Bayes, Elastic regularisation, kNN, generalised addictive models (GAM), Decision Tree, Random Forests, Neural Networks and SVM were employed to establish correlations between predictors and drug uptake. Tuning and hyper parameters search for each model were conducted through tenfold cross validation repeated 3 times on the training dataset; final model selection was based on minimisation of RMSE. The same training and test data set were employed for all models tested.

The best performing predictive model was used to estimate the drug uptake of the PBAE not previously experimentally tested in a two-steps approach. During the first round, amine and acrylates exhibiting a variety of structural features and moieties was employed to recognise critical patterns. In round2, variations of the pivotal characteristics observed in round1were explored to further refine the optimal candidate.

## Supplementary Information

Supplementary Information.

## Data Availability

The datasets used and/or analysed during the current study are available from the corresponding author on reasonable request.
