# Supplementary material for: Feasibility and application of machine learning enabled fast screening of poly-beta-amino-esters for cartilage therapies
Source: Sci Rep. 2022 Aug 20;12:14215. doi: 10.1038/s41598-022-18332-3 (PMC9392801; doi:10.1038/s41598-022-18332-3)
Supplement: Supplementary file 1 — Supplementary Information. [file 41598_2022_18332_MOESM1_ESM.docx]

Feasibility and Application of Machine Learning Enabled Fast Screening of Poly-beta-Amino-Esters for Cartilage Drug Therapies

Supplementary Info

*Stefano Perni, Polina Prokopovich*

School of Pharmacy and Pharmaceutical Sciences, Cardiff University, Cardiff, UK

Corresponding author: Dr Polina Prokopovich

School of Pharmacy and Pharmaceutical Sciences

Cardiff University

Redwood Building, King Edward VII Avenue

Cardiff, UK

CF10 3NB

E-mail address: prokopovichp@cf.ac.uk

# Additional Experimental methods

## Polymer synthesis

Acrylate-terminated poly(β-amino ester)s were obtained from diacrylate and amine monomers mixed in a 1.1:1 ratio. Acrylate (3.7 mmol) and amine (3.36 mmol) were dissolved in dichloro-methane (DCM) (5 ml); the polymerization was then performed under stirring at 50 °C for 48 hours. The reaction mixture was poured in diethyl-ether (50 ml) and the PBAE recovered removing the solvent under vacuum ^1^. Acrylate-terminated PBAEs were end-capped mixing with excess of end-capping agent ^1-2^.

## Conjugation of DEX to PBAEs

Dexamethasone (DEX) was succinylated (DEX-succ) using succinic anhydride in N,N-dimethyl-formamide (DMF) in the presence of 4-dimethylamino-pyridine (DMAP). The reaction mixture was kept under nitrogen for 24 h at room temperature with mixing; then the solvent was removed under vacuum ^1-2^. Purification of the prepared DEX-succ was carried out through repeated washing with dH_2_O ^1^.

DEX-succ was conjugated to amine end-capped PBAE in DCM containing N,N'-dicyclohexylcarbodiimide (DCC) and N-hydroxysulfosuccinimide (NHS). PBAEs-DEX conjugates were recovered in diethyl-ether followed by solvent evaporation under vacuum ^1-2^.

^1^H-NMR spectroscopy was performed (Bruker BioSpin GmbH) to identify the structures and estimate drug load of the conjugated PBAE–DEX. Samples were prepared at 10-12 mg/mL in DMSO-d_6_.

## Cartilage samples

Full depth explants of articular cartilage were excised under sterile conditions using a 6 mm diameter biopsy punches from the medial aspect of the medial condyle of individual metacarpo-phalangeal joints of bovine steers immature (7-day-old) feet obtained from a local abattoir ^1-2^.

## DEX uptake into cartilage using PBAE-DEX

Cartilage explants were cut in half, weighted and placed in one of the holding slots machined into a polytetrafluoroethylene (PTFE) transport chamber as previously described ^1-2^. Casein was employed to block non-specific binding of solutes to the chamber surfaces. The chamber space facing the cartilage outer surface was filled with PBS (50 μL) containing PBAEs-DEX at known concentration supplemented with protease inhibitors; the other chamber side was filled with PBS (50 μL) containing protease inhibitors alone ^1-2^.

The flow-chamber were incubated at 37 °C; evaporation was prevented placing the chamber in a Petri dish containing dH_2_O and covered. Stagnant layers at cartilage surfaces were prevented by placing the dish on a slow-speed rocker. Samples were removed at required intervals, washed in copious amount of water and placed in an Eppendorf containing digestion buffer (1 ml) ^1-2^. Experiments were performed on triplicate samples originated from 3 different animals ^1-2^.

Drug uptake achieved by each PBAE-DEX was compared to the dexamethasone phosphate (DEX-P) at the advised concentration of 4.4 mg/mL, equivalent to 4 mg/mL of DEX.

## Cartilage digestion

Cartilage samples were digested at 50 °C for 24 hours using 300 mg/L of papain dissolved in a phosphate buffer 0.2 M at pH = 6.8 containing, EDTA 1 mM and [dithiothreitol](http://en.wikipedia.org/wiki/Dithiothreitol) (DTT) 2mM.

## DEX quantification

Dexamethasone in the digestion buffer was quantified using rp-HPLC (Agilent, 1100 HPLC system). Separation was carried out with a TeknoKroma TRACE EXCEL 120 ODSB 5 μm analytical column maintained at 25°C. The injection volume was 25 μl, the mobile phase was 1 mL/min of a mixture of PBS:acetonitrile:glacial acetic acid 70:26:4; the detector was a UV spectrophotometer at 244 nm ^1-2^.

## Determination of chondrocytes viability in *ex-vivo* model

Cartilage samples were obtained as described above and equilibrated in serum free medium (low-glucose DMEM (Gibco, UK)), supplemented with 10 mM HEPES buffer (Invitrogen, CA), 1% ITS (containing insulin 10 μg/mL, transferrin 5.5 μg/mL and selenium 5 ng/mL), 0.1 mM non-essential amino acids, 20 μg/mL ascorbic acid, 100 units/mL penicillin G, 100 mg/mL streptomycin and 0.25 mg/mL amphotericin B for 2 days at 37 °C in 5% CO_2_.^2^

Samples (n = 6) were incubated at 37 °C in a humidified atmosphere containing 5% CO_2_; media was also supplemented with PBAE-DEX equivalent to 4 mg/ml of DEX or the equivalent amount of PBAE without conjugated DEX or 4.4 mg/ml of DEX-phosphate (corresponding to 4 ml/ml of DEX).

After an incubation period of 1 day or days, cartilage explants were washed in PBS and the mitochondrial activity of chondrocytes in the cartilage plugs was assessed through the MTT assay.


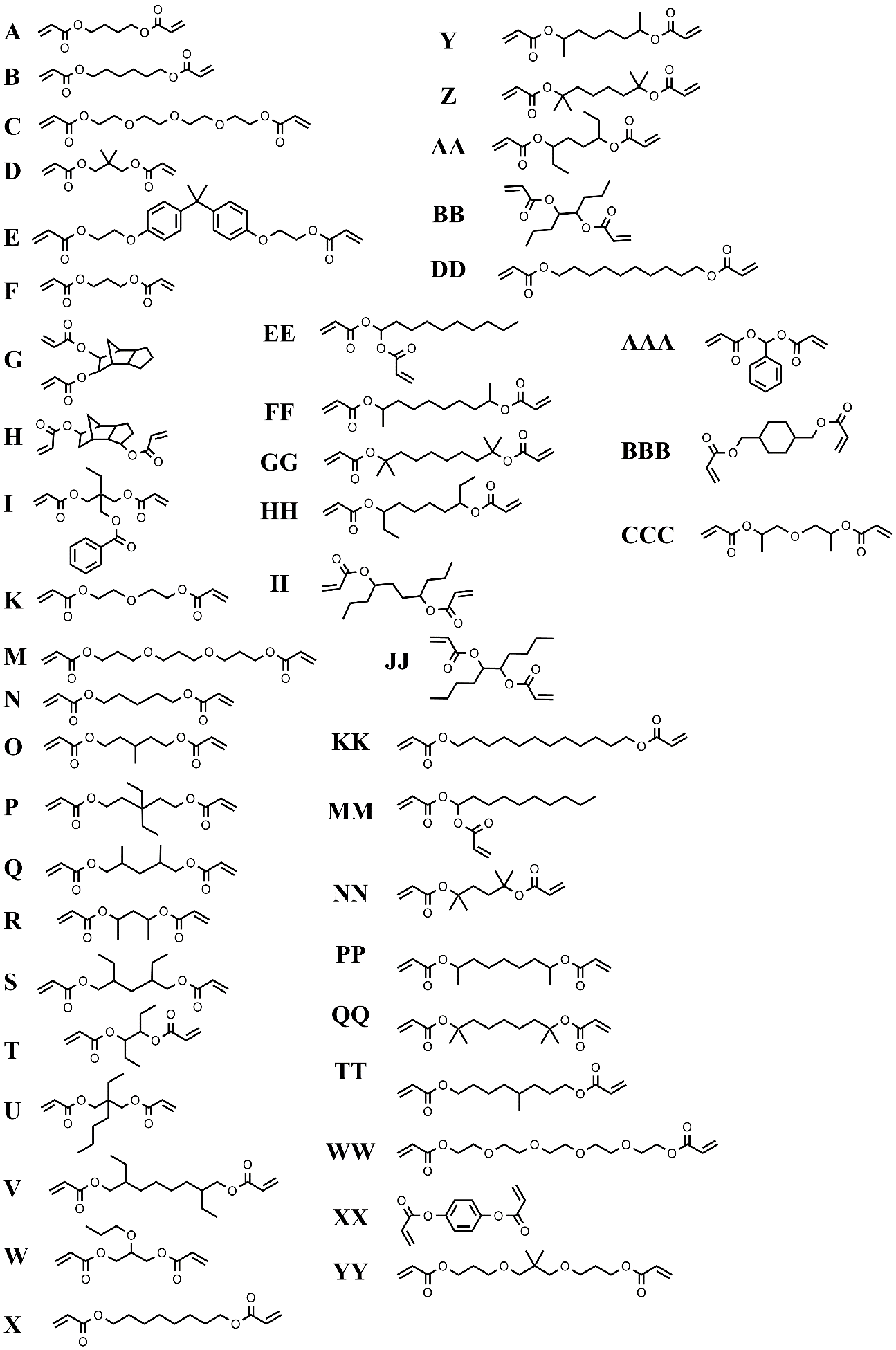


Figure S 1. Chemical structure and denomination of acrylates used.


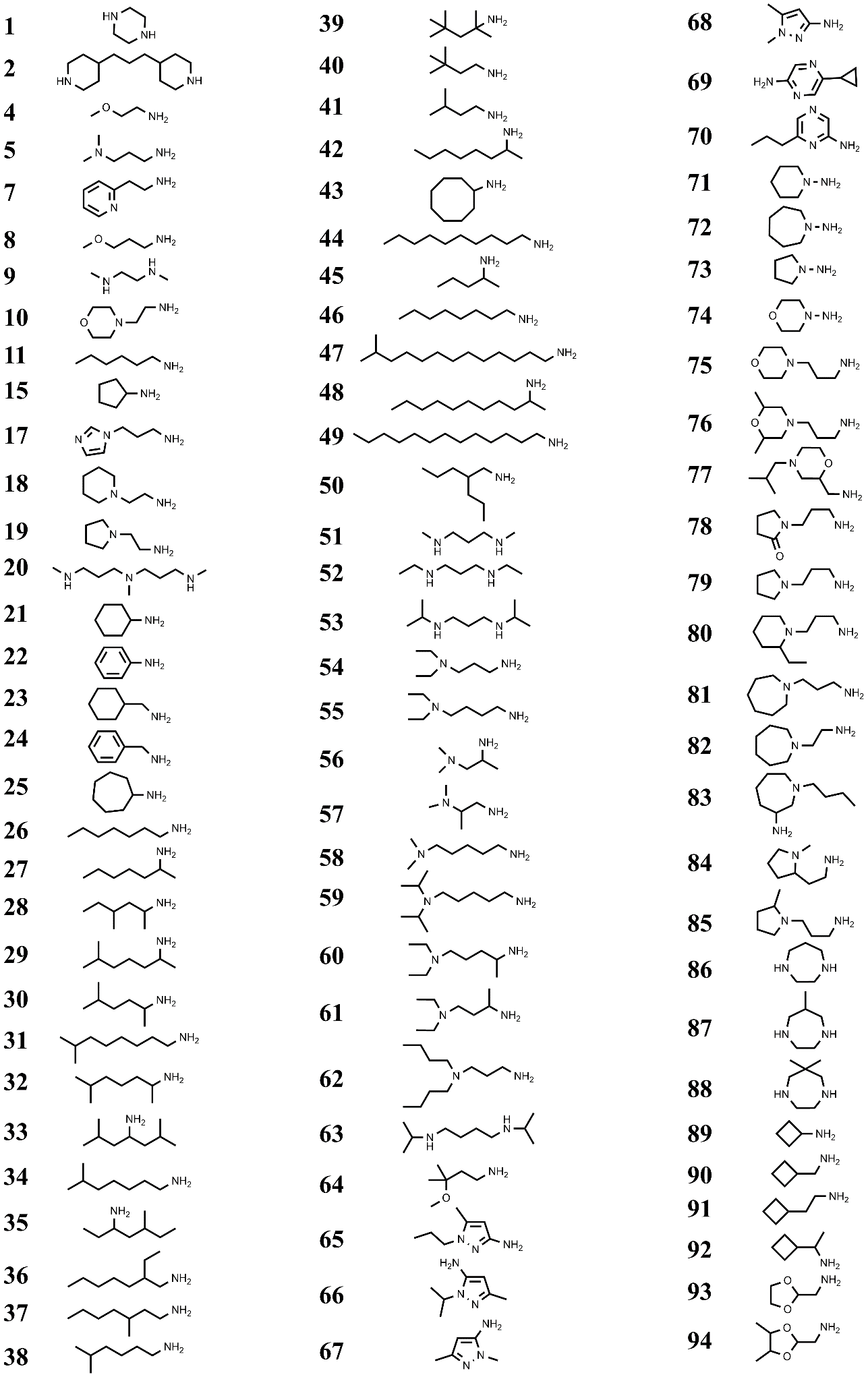


Figure S 2. Chemical structure and denomination of amines used.

# Additional results


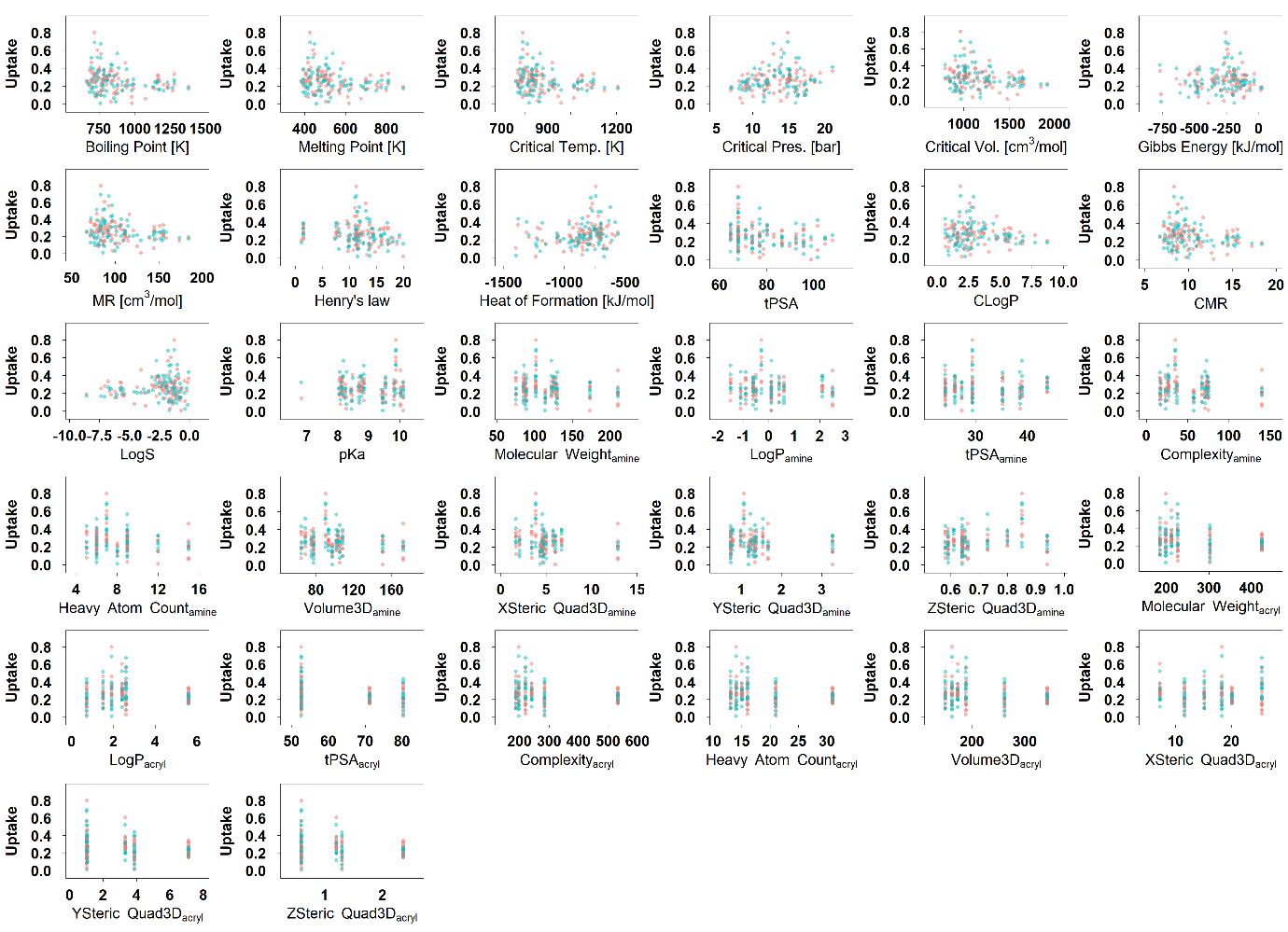


Figure S 3. Relation of experimental DEX uptake for PBAE-DEX (endcapped with e-1 ■ and e-2 ■) and indivudual chemico-physical properties of PBAEs.


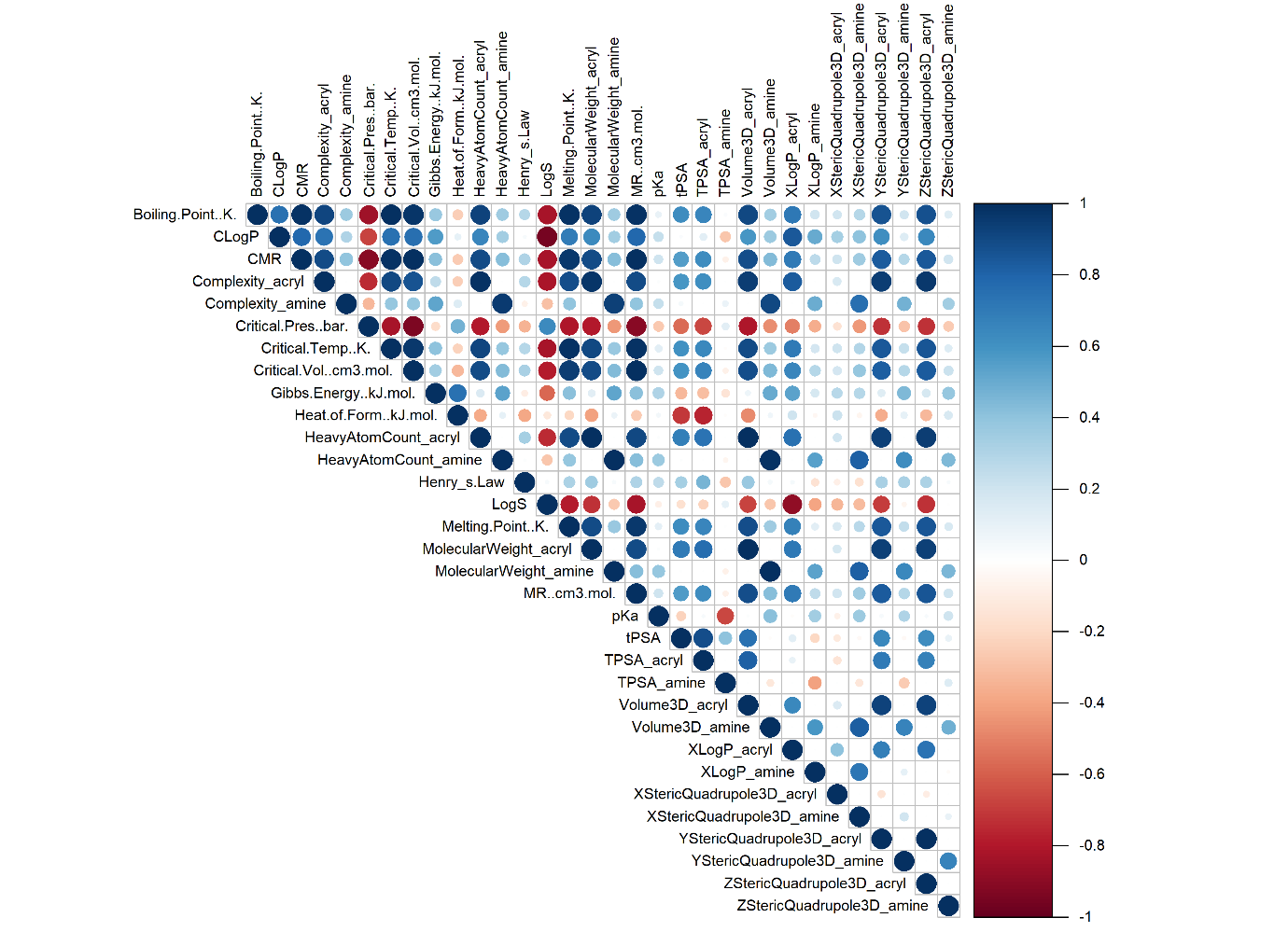


Figure S 4. Correlation matrix of the chemico-physical properties of the PBAE tested.


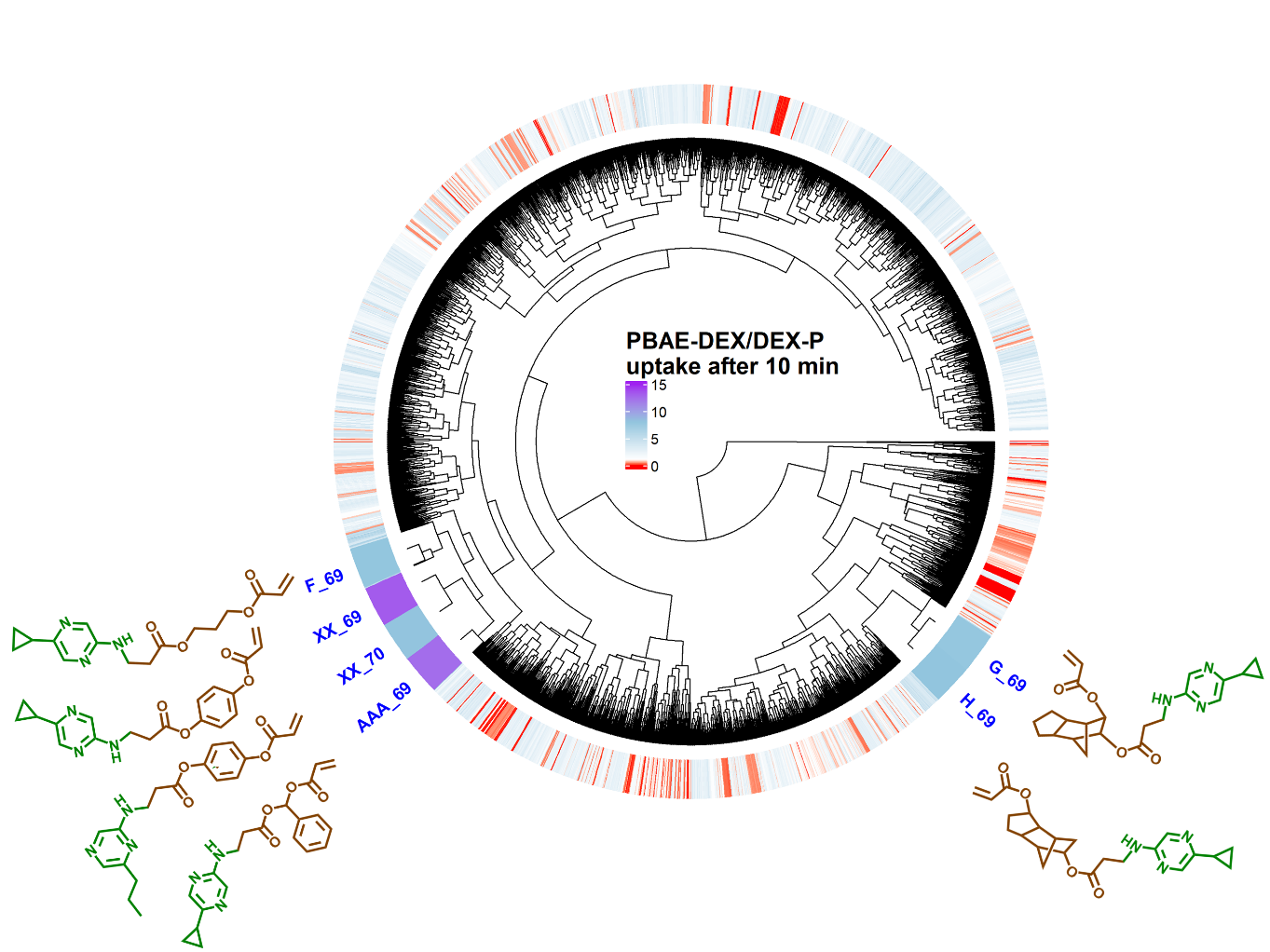


Figure S 5. Heatmap of predicted ratio of DEX uptake for PBAE endcapped with e2 conjugated with DEX over commercial formulation of DEX after 10 min of exposure and structure of PBAE repeated unit with predicted drug uptake superior to experimental found candidate.


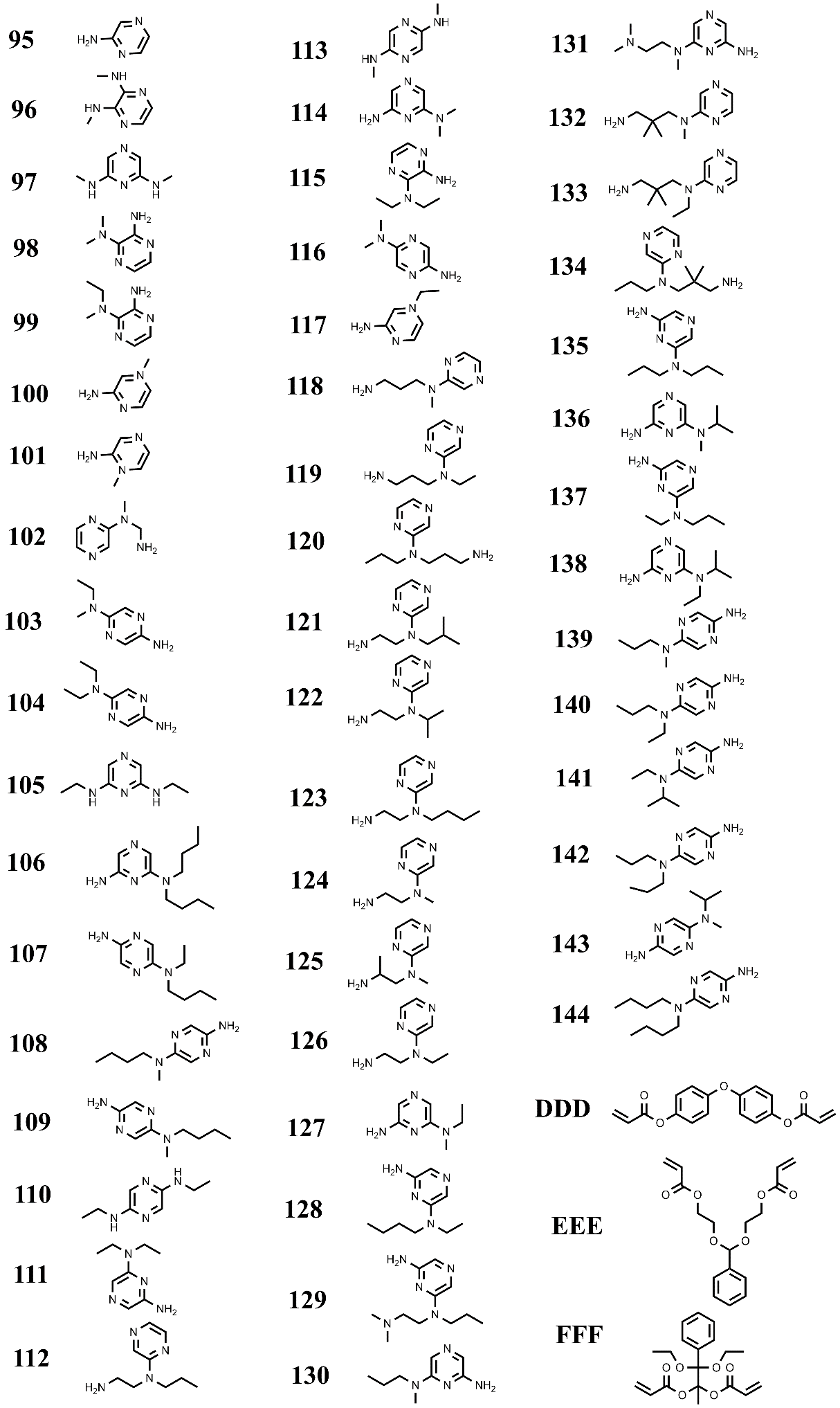


Figure S 6. Chemical structure and denomination of amines and acrylates used in round2.


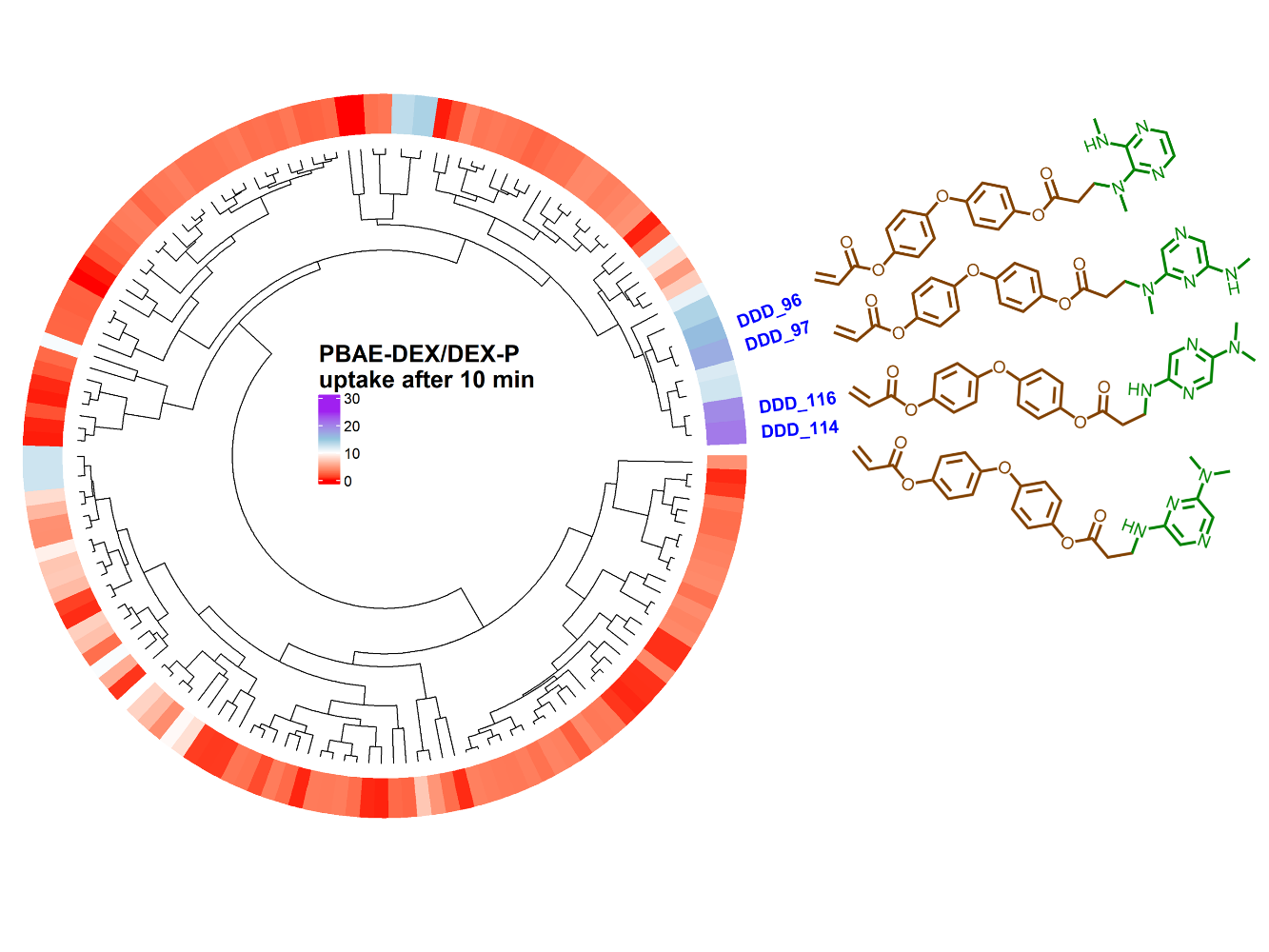


Figure S 7. Heatmap of predicted ratio of DEX uptake for PBAE during round2 endcapped with e2 conjugated with DEX over commercial formulation of DEX after 10 min of exposure and structure of PBAE repeated unit with predicted drug uptake superior to best candidate in round1.

# References

1. Perni, S.; Prokopovich, P., Poly-beta-amino-esters nano-vehicles based drug delivery system for cartilage. *Nanomedicine* **2017,** *13* (2), 539-548.

2. Perni, S.; Prokopovich, P., Optimisation and feature selection of poly-beta-amino-ester as a drug delivery system for cartilage. *J Mater Chem B* **2020,** *8* (23), 5096-5108.
